# Thymol as an Adjuvant to Restore Antibiotic Efficacy and Reduce Antimicrobial Resistance and Virulence Gene Expression in Enterotoxigenic *Escherichia coli* Strains

**DOI:** 10.3390/antibiotics11081073

**Published:** 2022-08-08

**Authors:** Andrea Bonetti, Benedetta Tugnoli, Andrea Piva, Ester Grilli

**Affiliations:** 1 Dipartimento di Scienze Mediche Veterinarie (DIMEVET), Università di Bologna, Via Tolara di Sopra 50, 40064 Ozzano dell’Emilia, Italy; 2 Vetagro S.p.A., Via Porro 2, 42124 Reggio Emilia, Italy; 3 Vetagro Inc., 17 E. Monroe St., Suite #179, Chicago, IL 60603, USA

**Keywords:** antibiotic resistance, enterotoxigenic *Escherichia coli*, piglets, thymol, post-weaning diarrhea, virulence genes, antibiotic resistance genes

## Abstract

The continuous spread of antimicrobial resistance is endangering the efficient control of enterotoxigenic *Escherichia coli* (ETEC), which is mainly responsible for post-weaning diarrhea onset in piglets. Thymol, the key constituent of thyme essential oil, is already used in animal nutrition for its antimicrobial action. The aim of this study was to investigate the potential adjuvant effect of thymol to re-establish antibiotic efficacy against highly resistant ETEC field strains. Secondly, we evaluated the modulation of virulence and antibiotic resistance genes. Thymol showed the capacity to control ETEC growth and, when combined with ineffective antibiotics, it increased their antimicrobial power. In particular, it showed significant effects when blended with colistin and tetracycline, suggesting that the adjuvant effects rely on the presence of complementary mechanisms of action between molecules, or the absence of resistance mechanisms that inactivate antibiotics and target sites. Furthermore, our findings demonstrate that, when added to antibiotics, thymol can help to further downregulate several virulence and antibiotic resistance genes, offering new insights on the potential mechanisms of action. Therefore, in a one-health approach, our study supports the beneficial effects of combining thymol with antibiotics to restore their efficacy, together with the possibility of targeting gene expression as a pioneering approach to manage ETEC pathogenicity.

## 1. Introduction

Following the continuous growth of the global population, FAO projections forecast a significant increase in total meat consumption by 2030 [1]. To meet such increasing demand, pig production farms will also need to optimize animal growth performance while ensuring the application of environmentally sustainable practices. This improvement can be achieved by supporting animal health in crucial periods such as weaning, a sudden phase during which piglets face a huge stress that can negatively influence the entire life of the animals [2,3].

Weaning stress often results in an impairment of the gastrointestinal tract and the overall health of piglets, with the consequent onset of post-weaning diarrhea (PWD) [4]. This debilitating condition is primarily caused by enterotoxigenic *Escherichia coli* (ETEC) infections [5]. Poor breeding conditions and bad management practices allow the persistence of the pathogen in the environment, which infects animals via the oral route [6]. ETEC strains exploit the intestinal weakness at weaning to overgrow and produce adhesins (e.g., F18 or F4) and toxins (e.g., heat-labile and heat-stable toxins) to finally target intestinal cells, causing diarrhea [7]. Costs for producers can become high, especially for the considerable reduction in growth performance and mortality [5].

Historically, PWD has been efficiently prevented thanks to the uses of high doses of zinc oxide. Such doses, ranging from 1000 to 3000 ppm in the feed, overcome nutritional requirements and are commonly referred as “pharmacological” [8,9]. This molecule controls ETEC infections by exerting a mild antibacterial action and reducing PWD symptoms through enhancement of the intestinal mucosa’s overall health [10]. However, pharmacological doses of zinc oxide are no longer allowed in the European Union from the end of June 2022 [10,11,12], so antibiotics remain—also under veterinary prescription—one of the few feasible and consolidated choices for full-blown disease treatment [4].

Nevertheless, the ever-expanding issue of antimicrobial resistance spread is endangering the powerful effects of antibiotics. Their use should be strictly limited to severe cases to protect antimicrobial efficacy for both veterinary and human medicine [13,14].

For this reason, alternative sources are attracting a growing amount of attention: studies have been directed towards the identification of novel natural matrices having antimicrobial properties, such as essential oils. The bioactive principles they contain can also be chemically synthesized to allow easier use in animal nutrition [15]. Amongst these, thymol is one of the most promising nature-identical compounds already authorized as a feed additive and has been extensively studied for its antimicrobial, antioxidant, and anti-inflammatory properties [16,17,18,19]. 

Thymol is an effective antimicrobial molecule against ETEC strains, and its power also extends to the modulation of virulence gene expression in pathogens [20]. Moreover, other studies have explored the option of combining antibiotics with nature-identical compounds, showing that they can be considered not only substitutes for but also auxiliary to commonly used antibiotics, even when resistance jeopardizes the antimicrobial effects [21,22,23,24].

Thus, the aim of this work was to assess thymol’s potential to work as an antibiotic adjuvant against highly resistant ETEC strains. Moreover, this study investigated how the combination of antibiotics and thymol modulated virulence and antibiotic resistance genes’ expression. 

## 2. Results

### 2.1. Minimal Inhibitory Concentration (MIC) Assay—Individual Compounds

#### 2.1.1. Antibiotics

The results of the MIC assay for antibiotics are reported in Table 1. ETEC were particularly resistant to a wide panel of antibiotics: at least half of the strains had a MIC higher than the reference breakpoint for amoxicillin, enrofloxacin, nalidixic acid, neomycin, trimethoprim, sulfamethoxazole, and tetracycline. However, other antibiotics showed an increasing pattern of resistance, since the MIC values were distributed across several sub-breakpoint concentrations as for florfenicol (16–4 mg/L) and the combination of amoxicillin with clavulanic acid (16/8–4/2 mg/L). Other antibiotics, such as colistin, showed bimodal trends, with some highly resistant strains and others being susceptible. Finally, the single antibiotic that was most efficient in controlling the growth of all the selected strains was ceftiofur, with MIC values always below the reference breakpoint.

#### 2.1.2. Thymol

**Figure 1:**
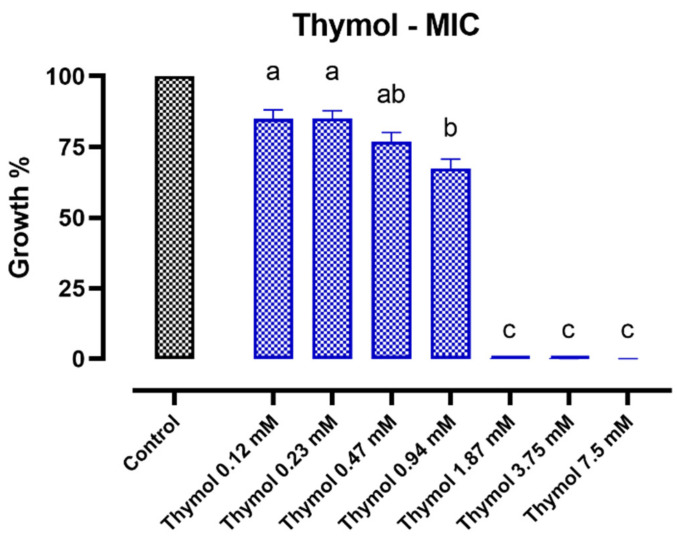
Growth of the six studied ETEC strains after 24 h of incubation with thymol. Growth is expressed as a percentage relative to the control (strain only); values are presented as means ± SEM of the mean growth of each strain for each concentration of thymol. Data were analyzed by one-way ANOVA with Tukey’s multiple comparison test; superscript letters (a, b, c) indicate significant differences among different doses (*p* < 0.05).

Figure 1 reports the cumulative results obtained for all the six ETEC strains when incubated with increasing concentrations of thymol. The MIC of thymol was reported at 1.87 mM for all the investigated bacterial strains. 

### 2.2. Minimal Inhibitory Concentration (MIC) Assay—Combinations

Antibiotics with more than half of the tested strains showing a high resistance pattern were then evaluated in combination with sub-MIC doses of thymol (half of the MIC and quarter of the MIC) to study potential adjuvant effects. The selected antibiotics were colistin, enrofloxacin, amoxicillin, tetracycline, trimethoprim with sulfamethoxazole, and neomycin. Colistin was added to the list because of its importance in veterinary and human medicine. For antibiotics that reported a MIC, three sub-MIC doses were evaluated (half of the MIC, quarter of the MIC, and one-sixth of the MIC), while for antibiotics that did not show a MIC value within the tested range of concentrations, the three highest doses were tested (64, 32, and 16 mg/L). For every antibiotic, all the resistant strains were tested, while for neomycin, only the three highly resistant strains (MIC > 64 mg/L) were chosen.

Table 2 shows the results of the inhibition of resistant ETEC strains when incubated with sub-MIC doses of colistin and/or thymol. The data showed that when 0.94 mM thymol was used (MIC/2), the efficacy of colistin was completely restored at the 16 mg/L dose (MIC/2), with partial effects registered also for the quarter of MIC (MIC/4) concentration, letting the antibiotic restore its efficacy at doses closer to the resistance breakpoint (4 mg/L). Equal results were also registered when sub-MIC doses of colistin (MIC/2 and MIC/4) were combined with 0.47 mM of thymol (MIC/4): the efficacy of the antibiotic was totally (100% inhibition of growth) or partially (47% inhibition) re-established. The same results were observed also for tetracycline (Table 3), but only at the highest dose of thymol (MIC/2) with the two highest tested concentrations of the antibiotic (64 and 32 mg/L); with thymol 0.47 mM, only minor numerical improvements were shown for the interaction with tetracycline.

Table 4, Table 5, Table 6 and Table 7 report the inhibition of ETEC strains resistant to trimethoprim with sulfamethoxazole, enrofloxacin, amoxicillin, and neomycin after incubation with sub-MIC concentrations of the antibiotics and/or thymol. The results show that, in all the displayed cases, sub-MIC doses of thymol are not able to significantly improve the activity of the antibiotics. Inhibition patterns are mainly driven by single compounds even if, in some cases mainly limited to trimethoprim/sulfamethoxazole and enrofloxacin, minor numerical improvements (*p* > 0.1) derived from the interaction of antibiotics and thymol were observed. 

### 2.3. Whole-Genome Sequencing (WGS) of ETEC 95 and ETEC 97

To elucidate the mechanism of action of the observed adjuvant effects between the antibiotics and thymol, the two strains ETEC 95 and ETEC 97, which are highly resistant to both colistin and tetracycline, were sequenced to select virulence and antibiotic resistance genes for the subsequent gene expression assay. The results of the characteristics obtained from the WGS analysis are reported in Table 8.

From a downstream analysis of the assembled draft genome sequences, ETEC 95 was revealed to belong to the O131:H4 serotype, while ETEC 97 was associated with the O138:H14 serotype. For both strains, the main fimbrial adhesin was F18, and the three toxins associated with the strains were the heat-stable toxins STa and STb, and the heat-labile toxin LT, which commonly characterize ETEC strains. The high pattern of resistance of the two strains is proved by the presence of a large number of resistance genes that target different classes of molecules, as shown in Table 8.

### 2.4. Gene Expression Analysis

#### 2.4.1. Virulence Genes

**Figure 2:**
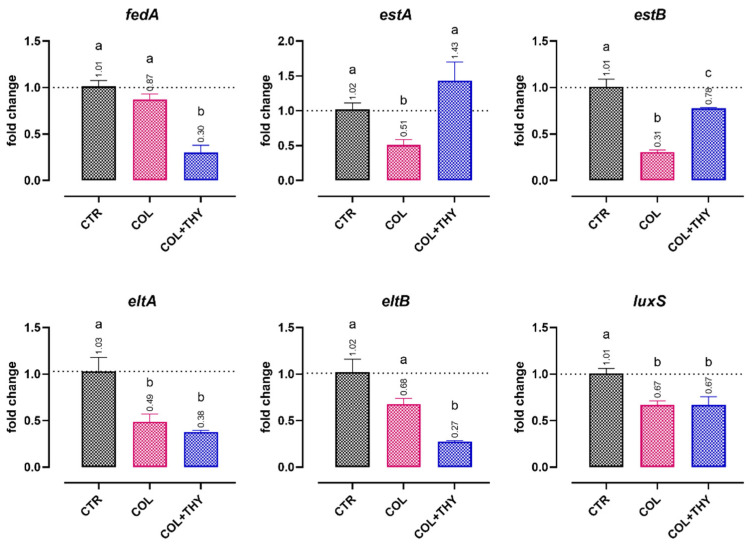
Effects of colistin (MIC/4, 8 mg/L, COL) or a combination of colistin and thymol (MIC/4, 0.47 mM, COL + THY) on ETEC 95′s and ETEC 97′s expression of virulence genes related to adhesion (*fedA*), heat-stable toxin expression (*estA* and *estB*), heat-labile toxin expression (*eltA* and *eltB*), and quorum sensing (*luxS*). Data are expressed as the means of the three technical replicates of the two studied strains, with the SEM reported as vertical bars. For each gene, the data were analyzed by one-way ANOVA with Tukey’s multiple comparison test; superscript letters (a, b, c) indicate significant differences among the groups (*p* < 0.05).

The expression of virulence genes of both the ETEC 95 and ETEC 97 strains when incubated with colistin (MIC/4, 8 mg/L) or a combination of colistin and thymol (MIC/4, 0.47 mM) is displayed in Figure 2. In general, the quarter-MIC dose of colistin downregulated the expression of all the selected virulence genes (*p* < 0.05), except for *fedA* and *eltB*, where only a numerical reduction was reported. The addition of thymol to the culture media of bacteria supported a further decrease in the expression levels of some virulence genes, as displayed by the results for *fedA* adhesin and the LT toxin subunit *eltB* (*p* < 0.05). In other cases, no substantial reductions were reported, even if the expression levels were maintained at values significantly lower than or equal to the control. 

**Figure 3:**
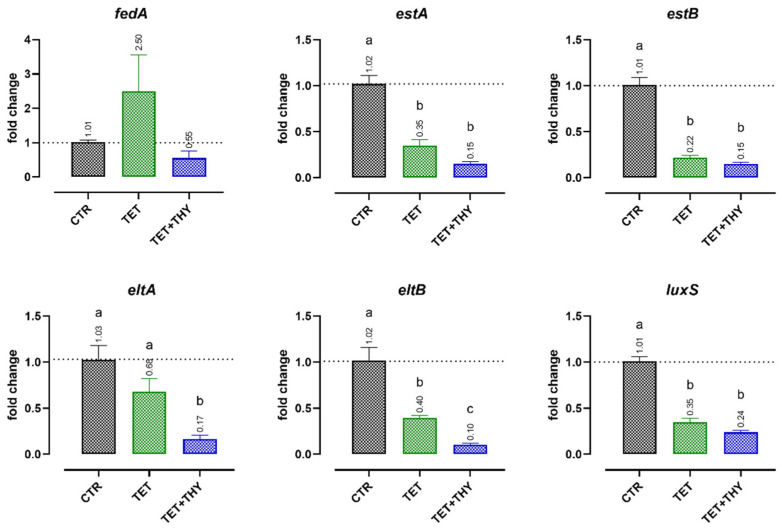
Effects of tetracycline (64 mg/L, TET) or a combination of tetracycline and thymol (MIC/4, 0.47 mM, TET + THY) on ETEC 95′s and ETEC 97′s expression of virulence genes related to adhesion (*fedA*), heat-stable toxin expression (*estA* and *estB*), heat-labile toxin expression (*eltA* and *eltB*), and quorum sensing (*luxS*). Data are expressed as the means of the three technical replicates of the two studied strains, with the SEM reported as vertical bars. For each gene, data were analyzed by one-way ANOVA with Tukey’s multiple comparison test; superscript letters (a, b, c) indicate significant differences among the groups (*p* < 0.05).

Figure 3 reports the results for the expression of virulence genes when ETEC 95 and ETEC 97 strains were incubated with tetracycline (64 mg/L) or the antibiotic with thymol (MIC/4, 0.47 mM). The utilization of tetracycline significantly helped in reducing the expression of all the studied virulence genes, with the unique exception of *fedA*. The gene that encodes the main subunit of the F18 adhesin reported a contrasting result for the two studied strains, with a reduction in its expression in the case of ETEC 97, and a considerable increase in mRNA levels for ETEC 95 (see also the Supplementary Material, Figure S1). However, the addition of sub-MIC doses of thymol to the antibiotic maintained or increased the downregulation of all genes. 

#### 2.4.2. Antibiotic Resistance Genes

**Figure 4:**
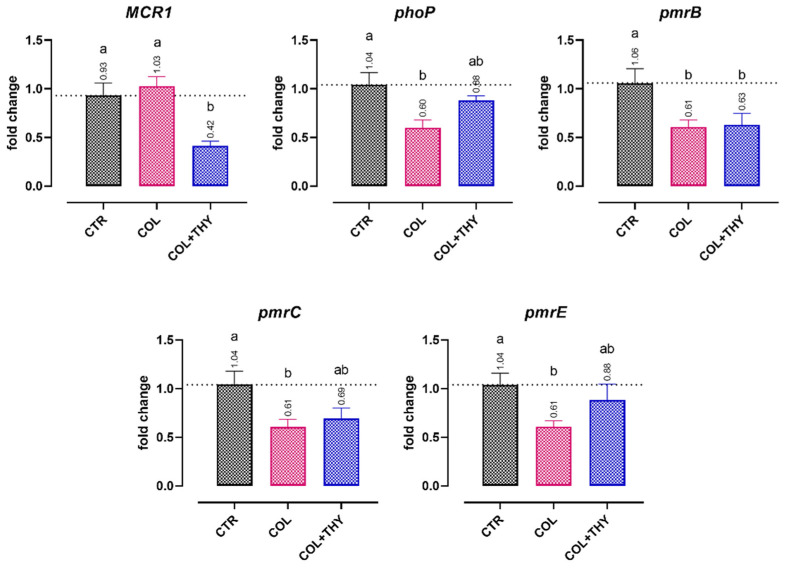
Effects of colistin (MIC/4, 8 mg/L, COL) or a combination of colistin and thymol (MIC/4, 0.47 mM, COL + THY) on ETEC 95′s and ETEC 97′s expression of antibiotic resistance genes related to enzymes that have a direct role in LPS modification (*MCR-1*, *pmrC*, and *pmrE*), and regulator proteins that belong to two-component systems (*phoP* and *pmrB*). Data are expressed as the means of the three technical replicates of the two studied strains, with the SEM reported as vertical bars. For each gene, the data were analyzed by one-way ANOVA with Tukey’s multiple comparison test; superscript letters (a, b) indicate significant differences between groups (*p* < 0.05).

The results for colistin-related antibiotic resistance gene expression are displayed in Figure 4, after the ETEC 95 and ETEC 97 strains, which are both highly resistant to colistin, were incubated with the antibiotic itself (MIC/4, 8 mg/L) or the antibiotic in combination with thymol (MIC/4, 0.47 mM). Colistin significantly reduced the expression of *phoP*, *pmrB*, *pmrC*, and *pmrE*, a group of chromosomal genes related to colistin resistance. The addition of thymol to colistin did not reduce the expression of the same genes any further. While colistin alone was not able to downregulate the RNA levels of the plasmidic resistance element MCR1, the addition of thymol significantly decreased the expression of the gene primarily responsible for colistin resistance.

**Figure 5:**
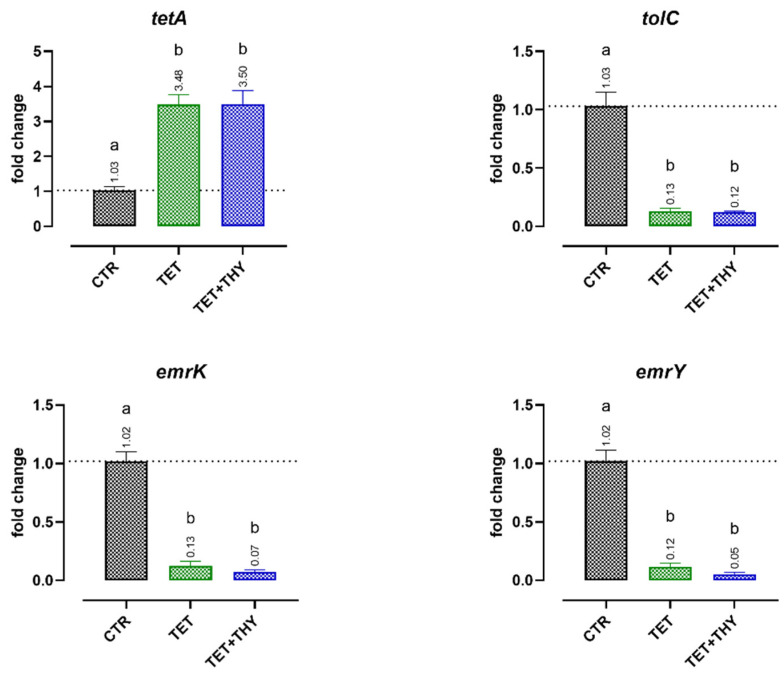
Effects of tetracycline (64 mg/L, TET) or a combination of tetracycline and thymol (MIC/4, 0.47 mM, TET + THY) on ETEC 95′s and ETEC 97′s expression of antibiotic resistance genes related to the production of tripartite efflux pumps (*emrK*, *emrY*, and *tolC*) and the specific tetracycline efflux pump (*tetA*). Data are expressed as the means of the three technical replicates of the two studied strains, with the SEM reported as vertical bars. For each gene, data were analyzed by one-way ANOVA with Tukey’s multiple comparison test; superscript letters (a, b) indicate significant differences between groups (*p* < 0.05).

Figure 5 reports the gene expression results obtained when ETEC 95 and ETEC 97 strains, which are both highly resistant to tetracycline, were incubated with tetracycline (64 mg/L) or the antibiotic in combination with thymol (MIC/4, 0.47 mM). Tetracycline significantly reduced the expression of the tripartite efflux pump formed by the *tolC*, *emrK*, and *emrY* genes. On the other hand, tetracycline increased the expression of *tetA*, a gene involved in antibiotic clearance from bacterial cells. Thymol did not further modulate the expression of all the selected genes.

## 3. Discussion

In the complex framework of piglets’ weaning stress and the elimination of pharmacological zinc oxide doses, the continuous spread of antimicrobial resistance is endangering the effectiveness of one of the primary control strategies for PWD [28]. As demonstrated by our results collected from antibiograms performed on field strains of ETEC, pathogens are rapidly becoming resistant to all the main classes of antibiotics. Not only are an expanding number of strains becoming resistant, but many bacteria are also acquiring an increasing amount of resistance determinants, as suggested by the very high MIC values, which were worryingly far from the MIC breakpoints. Our results are generally in agreement with the resistance patterns detected by surveillance programs in the European Union, with high resistance to sulfamethoxazole, trimethoprim, tetracycline, and amoxicillin; growing resistance traits—although still low—to nalidixic acid, chloramphenicol, and colistin; and sensitivity to third-generation cephalosporins [28]. In fact, ceftiofur did control the growth of all the tested strains: its resistance determinants, mainly *ampC* genes such as *blaCMY* cephalosporinases, were not detected in the two sequenced bacteria.

An analysis of the results by the antimicrobial mechanism of action showed that antibiotics that affect protein synthesis via inhibition of the 30S and 50S ribosomal subunits or by tRNA binding interference—such as amphenicols, aminoglycosides, and tetracyclines—are quickly losing efficacy. Only a few strains are still demonstrating susceptibility to florfenicol, though at a level which is drawing closer to the CLSI breakpoints, and these would already be considered resistant according to EUCAST. These resistance patterns are highly driven by the frequent colocalization of tetracycline resistance genes with other resistance determinants [29].

While quinolones and fluoroquinolones still have an ability to inhibit bacterial growth, as indicated by the susceptibility of half of the tested strains, antibiotics that target folic acid synthesis—such as trimethoprim and sulfamethoxazole—suffer from the spread of *drf* and *sul* resistance genes. This phenomenon is endangering the powerful combination of these two antibiotics, which historically played an important role in controlling bacterial infections linked to diarrheic symptoms such as traveler’s diarrhea in humans and PWD in piglets [29,30,31]. The result is also corroborated by the abundance of resistance determinants for sulphonamides and diaminopyrimidines inside the genomes of both the sequenced strains, ETEC 95 and ETEC 97.

Being one of the first-choice antibiotics, amoxicillin is worryingly losing its efficacy as well [4,32]. Its association with clavulanic acid restores antibiotic effectiveness by inhibiting beta-lactamases, even if certain strains are already showing a reversion to resistance [33,34,35,36]. More attention should also be paid to colistin, a molecule that mainly targets membrane integrity: some strains already display a high degree of resistance because of the dangerous spread of *MCR-1* [37,38], as also revealed by our WGS analysis. The reported resistance is of critical concern because this antibiotic is included in the list of highest priority critically important antimicrobials (CIAs), published by WHO [13].

Given the difficult circumstances, alternative molecules are urgently needed. An enormous reservoir of bioactive principles comes from nature, because plants need to naturally defend themselves from bacterial and viral infections [39,40,41]. Dry or oily extracts from plant sources are innately rich in antimicrobial principles, which can be isolated or chemically synthesized to obtain pure nature-identical compounds already authorized for animal nutrition [15]. Amongst them, thymol is one of the most powerful actors used to manage bacterial infections [19,42]. As confirmed by our findings, the growth of the ETEC strains can be effectively controlled by thymol. Interestingly, thymol reported the same MIC value for all the selected bacteria, independently from their resistance genetic background. 

It is demonstrated that thymol exerts its antimicrobial power by permeabilizing and depolarizing bacterial membranes thanks to its pore-forming activity [18,43]. Despite the similarity of its mechanism of action with several antibiotics, resistance determinants do not currently seem to affect thymol or other nature-identical compounds [44]. For this reason, the aim of this study was to investigate if thymol, as a representative of nature-identical compounds, could re-establish the efficacy of conventional antibiotics by exerting an adjuvant effect.

The results of the MIC tests against resistant strains showed that thymol can effectively support the action of antibiotics by significantly reducing the MIC and re-establishing their efficacy at doses closer to the MIC breakpoints. These outcomes are even more relevant if we consider that all the molecules were combined at sub-MIC concentrations, thus denoting the possibility to maintain a powerful action even when the antibiotic efficacy would be lost. 

However, thymol’s adjuvant effects against resistant strains of ETEC were registered only in a few selected cases. The first scenario occurred when the two chosen compounds showed a similar mechanism of action, as demonstrated by the results obtained with the synergistic combination of colistin and thymol. In fact, the two compounds have complementary effects, both directed against bacterial membranes. A putative explanatory mechanism of action could involve thymol as a main actor to damage the membranes; this disruption would expose LPS portions which could be further bound by colistin. This would facilitate antibiotic action independently from the lipid A modifications that usually protect the membranes from colistin itself [45].

Nevertheless, the synergistic action of thymol and colistin go, in all likelihood, beyond mechanistic effects on the membranes. Our results demonstrate that the combination of the two molecules can significantly reduce the expression of the *MCR-1* resistance determinant, a phosphatidylethanolamine transferase which alters the net charge of LPS by making the antibiotic ineffective [46]. The downregulation of *MCR-1* can reduce LPS modification, letting colistin become more effective, with the synergistic help of thymol, as already demonstrated for eugenol [47].

Since other chromosomal resistance genes, such as the *phoP* or *pmr* genes, were not significantly affected by the addition of thymol to colistin, it seems that the positive effect is limited to mobile plasmidic genetic elements. Surprisingly, for chromosomal resistance determinants, colistin shows a downregulating effect. This could be explained by the negative feedback mechanism that tightly regulates the *phoPQ* and *pmrAB* two-component system operons [48]: after the addition of the antibiotic and the initial stimulation of such genes, the negative regulator *mgrB* is triggered to repress the activity and expression of *phoPQ* [49,50]. Because *pmr* genes are under the control of the *phoPQ* response as well, the downregulation also inevitably affects their expression [49]. Interestingly, the addition of thymol to colistin, other than restoring antibiotic efficacy, maintained the overall downregulating effect on such resistance genes, as happens for other compounds [51]. 

As already reported in our previous study, colistin at sub-MIC doses can efficaciously control the pathogenicity of *E. coli* K88 by reducing virulence gene expression [20]. This appears to also be confirmed for the highly resistant strains in our present study. With the addition of thymol to the system, the effects of colistin on plasmid-encoded ETEC virulence factors was further amplified. Amongst the analyzed genes, thymol produced an additional significant reduction in the expression of genes related to LT toxin and the *fedA* subunit of F18 adhesin. The latter is of particular interest because adhesion is a pivotal step for ETEC pathogenicity, and the synthesis of toxins is greatly stimulated after close bacterial interaction with the target cells [7,52,53].

The second case in which adjuvant effects are registered occurs when the resistance mechanism consists of the removal of the antibiotic from the bacterial cytoplasm. This is the case for tetracycline because resistance is mainly related to the acquisition of *tet* genes that encode for efflux pumps [54,55]. When these channels are activated by tetracycline, the addition of sub-MIC doses of thymol supports the action of the antibiotic. Since thymol is known to create pores on the surface of bacteria, it can re-establish tetracycline’s efficacy by helping it to enter inside the cells, acting against the action of pumps like *tetA*, carried by both the sequenced strains of this study. Moreover, thymol is also known to directly inhibit efflux pumps’ activity [17,56,57,58].

In general, the data showed that thymol’s supportive action on tetracycline mainly takes place through the modulation of antibiotic movement across bacterial membranes rather than directly affecting the genetic expression of efflux pumps. As a confirmation, *tetA* was not influenced by the addition of thymol, and no ameliorative effects were registered for the *emrKY-tolC* system, a multidrug efflux pump [59]. In particular, the expression of the chromosomal *emrKY-tolC* genes is tightly regulated by *phoPQ-* and *evgAS*-sensing systems and is influenced by several negative feedback mechanisms [59,60]. It is possible that, as already discussed for colistin’s chromosomal resistance determinants, in our culture conditions, ETEC bacteria experienced downregulation of such efflux pumps after their initial activation, an effect that would be maintained with thymol.

When resistance systems do not inactivate the antibiotic, the small amounts that can still enter resistant bacteria are capable of somewhat reducing ETEC virulence, as shown by the outcomes of this study when tetracycline was used. This phenomenon is probably dependent on the effective resistance level of the studied strains, as happened with *fedA* in the presence of tetracycline alone: while one strain experienced downregulation of the virulence gene, the other registered a significant increase in mRNA levels. However, with the addition of thymol, virulence genes underwent—in any case—an additional reduction in their expression. This is a supplementary confirmation of the mechanism of action of thymol, which helps tetracycline to remain inside bacterial cells, acting against *tetA* action [61]. The antibiotic can then impair ribosomal transcription, also affecting the expression of many ETEC virulence genes involved in the synthesis of adhesins, toxins, and quorum-sensing determinants.

When, amongst the resistance mechanisms, there is complete inactivation of the antibiotic or the substitution of the target, as occurs with trimethoprim, sulfamethoxazole, enrofloxacin, neomycin, and amoxicillin, the results do not show increased efficacy when thymol is added to the antibiotic, thus suggesting that the preservation of an active form of the antimicrobial and/or an intact site of action is essential for carrying out the adjuvant effect.

This novel approach of potentiating antibiotic effects appears to break the commonly-established rule of an increase in virulence when many resistance determinants are acquired [62,63]. In fact, our results clearly show that the adjuvant effect of thymol reduces, in most cases, both the antibiotic resistance and virulence gene expression of ETEC strains, while simultaneously increasing antibiotic efficacy and re-establishing newer MICs at values closer to the resistance breakpoints. The outcomes suggest the influence on transcription patterns as an additional mechanism of action of the beneficial effect of complexing sub-MIC doses of nature-identical compounds with antibiotics [64]. In a global context of antimicrobial resistance spread, our findings propose a way to restore and preserve antibiotics’ efficacy not only against PWD and ETEC infections in piglets, but also for human medicine, in a One Health perspective. 

## 4. Materials and Methods

### 4.1. Bacterial Strains and Culture Conditions

The six bacteria strains used in this study all belonged to the group of enterotoxigenic *Escherichia coli* (ETEC, Table 9) and were obtained from Istituto Zooprofilattico Sperimentale della Lombardia e dell’Emilia-Romagna (IZSLER), Reggio Emilia, Italy. Bacteria were all isolated from the feces or intestines of piglets with clinical signs of post-weaning diarrhea, and had been characterized by IZSLER as ETEC strains. Upon reception, the lyophilized cultures were activated and routinely cultured in brain–heart infusion broth (BHI; VWR International Srl, Milan, Italy) (pH 6.5) at 37 °C under aerobic conditions and counted via plating 10-fold serial dilutions onto BHI agar. Daily 1:100 passage was performed to maintain an active culture, or cultures were frozen for long-term storage.

### 4.2. Chemicals and Stock Solutions

The antibiotics (AB) tested in this study were amoxicillin, amoxicillin with clavulanic acid (2:1), ceftiofur, colistin, trimethoprim with sulfamethoxazole (1:19), nalidixic acid, enrofloxacin, florfenicol, neomycin, and tetracycline (all obtained from Alpha Aesar, Thermo Fisher GmbH, Kandel, Germany). AB stock solutions were prepared fresh in BHI or ethanol 100%, depending on the antibiotics’ solubility. Combinations of antibiotics were used at the ratio suggested by the CLSI guidelines. Thymol was obtained from Merck KGaA (Darmstadt, Germany), and the stock was prepared in 70% ethanol at a concentration that would ensure a final ethanol amount of ≤3.5% in the final dilutions. Each solution was filter-sterilized and diluted in sterile BHI to reach the final concentration tested.

### 4.3. Minimal Inhibitory Concentration (MIC) Assay—Individual Compounds

The MICs of AB and thymol were determined using the microdilution method in 96-well microtiter plates. Each of the tested strains was tested against a wide range of concentrations of all the selected antibiotics and thymol (Table 10). The bacterial strains (10^5^ CFU/mL) were incubated with the tested substances at 37 °C for 24 h under aerobic conditions. Control strains were grown in BHI supplemented with 3.5% ethanol to exclude inhibitory effects exerted by the ethanol finally contained in the stock solutions. After incubation, absorbance was read at a wavelength of 630 nm with a spectrophotometer (Varioskan LUX Multimode Microplate Reader, Thermo Fisher Scientific Inc., Waltham, MA, USA) to measure bacterial growth. The MIC value was defined as the lowest concentration of each compound which gave null absorbance (i.e., the bacterial growth) after an incubation period of 24 h.

### 4.4. Minimal Inhibitory Concentration (MIC) Assay—Combinations

After the screening of single molecules, resistant ETEC strains were incubated with combinations of thymol and some of the previously assessed AB at sub-MIC doses in a 3 × 2 checkerboard design. Antibiotics were chosen amongst the ones showing a high resistance pattern or because of their importance in animal care, with particular interest regarding piglets. The selected AB were amoxicillin, colistin, trimethoprim with sulfamethoxazole, enrofloxacin, neomycin, and tetracycline (Table 11). The three highest sub-MIC doses of each selected AB were combined with two sub-MIC doses of thymol, that is 0.94 and 0.47 mM. The MIC experiment, including incubation and absorbance measurements, were performed as described for the assay of individual compounds.

### 4.5. Whole-Genome Sequencing (WGS) and Sequence Analysis of ETEC 95 and ETEC 97

As two of the most resistant strains, ETEC 95 and ETEC 97 were selected for WGS analysis to find antibiotic and virulence resistance genes that would be useful for the subsequent gene expression study. An overnight BHI–agar plate of each of the two selected strains was sent to ISZLER, Parma, Italy, for total DNA extraction, quality check, and subsequent WGS. Sequencing was performed with Illumina MiSeq (Illumina Inc., San Diego, CA, USA) with paired-end sequencing (2 × 250 bp) and an average coverage of 100×. Genome sequences were assembled with UniCycler v.0.4.9 [65] and annotated using the PATRIC webtool v.3.6.12 with the RAST toolkit [66].

Thanks to the tools provided by the Center of Genomic and Epidemiology (CGE) of the National Food Institute of Denmark and the Technical University of Denmark [67], the presence of resistance genes (ResFinder tool) and virulence-associated genes (VirulenceFinder) were further confirmed, and the sequences of interest were extracted for primer design. Moreover, the serotype (SeroTypeFinder) and plasmid incompatibility groups (PlasmidFinder) were also evaluated with CGE tools.

### 4.6. Gene Expression Analysis

To assess how virulence and antimicrobial resistance genes are affected by the combinations of antibiotics with thymol in resistant strains, a gene expression analysis was performed by RT-qPCR. This analysis was performed on bacterial cells of ETEC 95 and ETEC 97 strains adapted in a growth medium (BHI) containing combinations of both antibiotics and thymol. The two strains were chosen for being the only two bacterial strains of the analyzed panel to have resistance against both colistin and tetracycline. The two antibiotics were selected for the gene expression analysis because of their importance in both veterinary and human medicine, and for being the ones for which the highest interaction was measured for the MIC in the combination.

To prepare adapted bacteria for the gene expression analysis, 10^6^ CFU/mL of overnight ETEC cultures of both selected strains were grown for 4 h at 37 °C after inoculation into new tubes with 5 mL of fresh BHI supplemented with AB alone or AB with thymol. The 0.47 mM dose of thymol was selected (MIC/4, quarter of MIC), while for AB, the 64 mg/L dose was chosen for tetracycline (MIC not found), and the 8 mg/L dose (MIC/4) was selected for colistin.

Bacterial RNA extraction and retrotranscription were performed as previously described [20,68]. Briefly, after incubation, tubes were centrifuged for 5 min at 5000× *g*, the supernatant was discarded, and the pellet was incubated for 10 min at 37 °C in a Tris-EDTA buffer with 1 mg/mL lysozyme. Total RNA extraction was conducted with the NucleoSpin RNA Kit (Macherey-Nagel GmbH & Co. KG, Düren, Germany), and DNase digestion was performed following the manufacturer’s instructions. RNA yield and quality were verified spectrophotometrically (μDrop Plate and Varioskan LUX, Thermo Fisher Scientific Inc., Waltham, MA, USA). For this, 800 ng of RNA was subsequently reverse-transcribed with an iScript cDNA Synthesis Kit (Bio-Rad Laboratories, Inc., Hercules, CA, USA) according to the manufacturer’s instructions. 

The obtained cDNA was used in the real-time PCR (qPCR) analysis. Amplifications were performed in a final volume of 10 μL, containing 5 μL of 2× iTaq Universal SYBR Green Supermix (Bio-Rad Laboratories, Inc., Hercules, CA, USA), 200 or 600 nM of each forward and reverse primer, 2 μL of 5 ng/μL cDNA, and nuclease-free water up to the full volume. Real-time PCR was performed using a CFX96 Real-Time PCR Detection System (Bio-Rad Laboratories, Inc., Hercules, CA, USA) under the following conditions: 3 min at 95 °C, followed by 40 cycles of 95 °C for 10 s and 60 °C for 30 s. The specificity of each reaction was evaluated by melting curve analysis with a 0.5 °C/s heating rate from 55 up to 95 °C. 

The expression of all the selected genes was normalized according to two reference genes, namely the 16S rRNA gene (16S) and the B subunit of the integration host factor (*ihfB*), as previously optimized in our laboratories. Relative changes in gene expression were calculated according to the 2^−ΔΔCt^ method [69].

Forward (F) and reverse (R) primers (Table 12) were designed or verified for alignment on the sequences of the genes of interest extracted from the WGS of ETEC 95 and ETEC 97 using the Primer-BLAST tool (NCBI; National Center for Biotechnology Information) and synthesized by Merck KGaA (Darmstadt, Germany).

### 4.7. Statistical Analysis

For the MIC assays (single compounds), for both AB and thymol, experiments were performed in two technical replicates for each strain. For AB, the reported values represent the lowest concentration of the substance that inhibited bacterial growth, while those for thymol values are presented as means ± SEM for all the six investigated strains. Bacteria were considered sensitive or resistant to AB according to the CLSI and EUCAST breakpoints [25,26,27].

For MIC assays (combinations), experiments were performed in three technical replicates for each assessed combination for each strain. Data in the tables are presented as means. Differences were analyzed with GraphPad Prism v.9.3.1 (GraphPad Software, Inc., San Diego, CA, USA) by performing one-way ANOVA with Sidak’s multiple comparisons test to investigate significant differences between single molecules and combination groups.

For the gene expression analysis, each experimental group was assessed in triplicate for both ETEC 95 and ETEC 97, and the results are presented as means ± SEM. Gene expression data were analyzed with GraphPad Prism v.9.3.1, performing one-way ANOVA with Tukey’s multiple comparisons test.

Differences were considered significant at *p* ≤ 0.05.

## 5. Conclusions

Our data show that although antibiotics are quickly losing their efficacy because of the ever-growing spread of antimicrobial resistance, nature-identical compounds such as thymol can effectively control the growth of enterotoxigenic *Escherichia coli* (ETEC). When sub-MIC doses of thymol are combined with antibiotics that show high resistance patterns, their action can be re-established and the MIC lowered at doses closer to the breakpoints.

However, the potential adjuvant effect of thymol appears to be dependent on the maintenance of antibiotic integrity and/or the target structure. That is, thymol’s synergism is particularly evident when it has a complementary mechanism of action to the antibiotics or it reverses the effects of resistance genes.

Our study also demonstrated that the combination of thymol with antibiotics is not merely related to the mechanistic interaction of the two classes of compounds, but also to a significant effect on the expression of several resistance and virulence genes.

These outcomes can contribute to further deepen our knowledge on the adjuvant effects and to develop innovative therapeutic approaches, other than supporting the modulation of gene expression as a new way to target bacterial growth, both for animal and human health.

## Figures and Tables

**Table 1:** Frequency of MIC values of 12 antibiotics (or combinations of antibiotics) against six field strains of ETEC. Vertical bars indicate MIC breakpoints as reported by EUCAST and CLSI [25,26,27]; values indicate the number of strains which reported a precise MIC value.

Enterotoxigenic *Escherichia coli* (ETEC)									
Antibiotics	MIC (mg/L)								
	>64	64	32	16	8	4	2	1	0.5
Amoxicillin	5						1		
Ceftiofur							1	4	1
Colistin			2					2	2
Nalidixic acid	3					3			
Enrofloxacin							3		3
Florfenicol	1			3	1	1			
Neomycin	3	1	2						
Trimethoprim	2	2							2
Tetracycline	4							2	
	>608	608	304	152	76	38	19	9.5	4.75
Sulfamethoxazole	4		1	1					
	>64/32	64/32	32/16	16/8	8/4	4/2	2/1	1/0.5	0.5/0.25
Amox./Clav. (2:1) ^1^			1	2	2	1			
	>32/608	32/608	16/304	8/152	4/76	2/38	1/19	0.5/9.5	0.25/4.75
Trim./Sulf. (1:19) ^2^	4								2

^1^ The combination of amoxicillin (Amox.) with clavulanic acid (Clav.) was tested at a 2:1 ratio as suggested by the CLSI guidelines. ^2^ The combination of trimethoprim (Trim.) with sulfamethoxazole (Sulf.) was tested at a 1:19 ratio as suggested by the CLSI guidelines.

**Table 2:** Percentage inhibition of the two ETEC strains resistant to colistin in the presence of combinations of colistin (COL, mg/L) and thymol (THY, mM).

Inhibition		COL 16	COL 8	COL 4
	%	35	10	7
THY 0.94	15	100 *§	54 *§	35
THY 0.47	8	100 *§	47 *§	38

* Significant difference between the combination and thymol alone. § Significant difference between the combination and colistin alone.

**Table 3:** Percentage inhibition of the four ETEC strains resistant to tetracycline in the presence of combinations of tetracycline (TET, mg/L) and thymol (THY, mM).

Inhibition		TET 64	TET 32	TET 16
	%	53	35	28
THY 0.94	47	95 *§	77 *§	61 *
THY 0.47	32	61	47	39

* Significant difference between the combination and thymol alone. § Significant difference between the combination and tetracycline alone.

**Table 4:** Percentage inhibition of the five ETEC strains resistant to trimethoprim + sulfamethoxazole in the presence of combinations of the two antibiotics (T + S, mg/L) and thymol (THY, mM).

Inhibition		T + S 32/608	T + S 16/304	T + S 8/152
	%	39	25	19
THY 0.94	25	45 §	34	30
THY 0.47	22	41 §	30	25

§ Significant difference between the combination and trimethoprim + sulfamethoxazole alone.

**Table 5:** Percentage inhibition of the three ETEC strains resistant to enrofloxacin in the presence of combinations of enrofloxacin (ENR, mg/L) and thymol (THY, mM).

Inhibition		ENR 1	ENR 0.5	ENR 0.25
	%	53	24	17
THY 0.94	38	79 §	46	38
THY 0.47	25	48 §	30	25

§ Significant difference between the combination and enrofloxacin alone.

**Table 6:** Percentage inhibition of the five ETEC strains resistant to amoxicillin in the presence of combinations of amoxicillin (AMO, mg/L) and thymol (THY, mM).

Inhibition		AMO 64	AMO 32	AMO 16
	%	9	14	16
THY 0.94	25	15	26	27
THY 0.47	21	15	24	25

**Table 7:** Percentage inhibition of the three ETEC strains resistant to neomycin in the presence of combinations of neomycin (NEO, mg/L) and thymol (THY, mM).

Inhibition		NEO 64	NEO 32	NEO 16
	%	6	8	8
THY 0.94	16	11	10	10
THY 0.47	10	9	13	12

**Table 8:** Characteristics of ETEC 95 and ETEC 97 strains selected for WGS and gene expression analysis.

Strain		ETEC 95	ETEC 97
Serotype		O131:H4	O138:H14
Adhesin		F18	F18
Toxins		STa, STb, LT	STa, STb, LT
Other virulence genes		*fedA*, *fedF*, *gad*, *hra*, *iha*, *iss*, *neuC*, *ompT*, *terC*, *traT*	*air*, *astA*, *cba*, *chuA*, *cma*, *fedA*, *fedF*, *iss*, *lpfA*, *ltcA*, *ompT*, *terC*, *traT*
Plasmids		IncFII(29), IncHI2, IncHI2A, IncQ1, IncX1	IncFIB, IncFII, IncI1-I, IncX1, IncX4
Resistance genes	Aminoglycosides	*aadA1*, *aph(3′)-Ia*, *aph(3′’)-Ib*, *aac(3)-IIa*, *aph(6)-Id*	*aac(3)-IV*, *aph(3′)-Ia*, *aph(4)-Ia*, *aadA2*, *aadA1*
	Polymyxins	*MCR-1*, *pmrC*, *pmrE*, *pmrF*	*MCR-1*, *pmrC*, *pmrE*, *pmrF*
	Folate pathway	*sul1*, *sul2*, *sul3*, *drfA1*	*sul3*, *drfA12*
	Tetracyclines	*tet(A)*, *emrK*, *emrY*, *tolC*	*tet(A)*, *emrK*, *emrY*, *tolC*
	Quinolones	*gyrA*, *emrK*, *emrY*, *tolC*	*gyrA*, *emrK*, *emrY*, *tolC*
	Amphenicols	*marA*, *marB*, *marR*	*cmlA1*, *floR*
	Beta-lactams	*blaTEM-1A*	*blaTEM-1A*
Observed antimicrobial resistance (MIC in mg/L) ^1^		AMO (>64), AMO + CLA (16/8), COL (32), TRI (>64), SUL (>608), TRI + SUL (>32/608), NAL (>64), ENR (2), FLO (16), NEO (>64), TET (>64)	AMO (>64), AMO + CLA (32/16), COL (32), TRI (>64), SUL (>608), TRI + SUL (>32/608), NAL (>64), ENR (2), FLO (>64), NEO (>64), TET (>64)

^1^ AMO, amoxicillin; AMO + CLA, amoxicillin with clavulanic acid (2:1); COL, colistin; TRI, trimethoprim; SUL, sulfamethoxazole; TRI + SUL, trimethoprim with sulfamethoxazole (1:19); NAL, nalidixic acid; ENR, enrofloxacin; FLO, florfenicol; NEO, neomycin; TET, tetracycline.

**Table 9:** Pattern of adhesins and toxins expressed by the six ETEC strains used in this study.

ETEC Strain	Adhesin	Toxins
ETEC 95	F18^+^	STa^+^; STb^+^; LT^+^
ETEC 97	F18^+^	STa^+^; STb^+^; LT^+^
ETEC 99	F4^+^	STa^+^; STb^+^; LT^+^
ETEC 104	F18^+^	STa^+^; STb^+^
ETEC 105	F4^+^	STa^+^; STb^+^
ETEC 106	F18^+^	STa^+^; STb^+^

**Table 10:** Range of tested concentrations for all the compounds analyzed in the MIC tests.

Molecule	Range of Tested Concentrations
Single antibiotics	0.5–64 mg/L
Amoxicillin/clavulanic acid (2:1) ^1^	0.5/0.25–64/32 mg/L
Trimethoprim/sulfamethoxazole (1:19) ^1^	0.25/4.75–32/608 mg/L
Sulfamethoxazole	608–4.75 mg/L
Thymol	0.12–7.5 mM

^1^ Ratios between the antibiotics were chosen according to the CLSI guidelines.

**Table 11:** Range of AB sub-MIC concentrations when analyzed in MIC tests in combination.

Antibiotic	Sub-MIC Tested Concentrations
Amoxicillin, neomycin, tetracycline	64–32–16 mg/L
Enrofloxacin	1–0.5–0.25 mg/L
Trimethoprim/sulfamethoxazole (1:19) ^1^	32/608–16/304–8/152 mg/L
Colistin	16–8–4 mg/L

^1^ Ratios between the antibiotics were chosen according to the CLSI guidelines.

**Table 12:** Primers used in this study for qPCR analysis.

Functions	Gene	Sequences (5′→3′)	Product Length (bp)	Ref ^1^
Adhesion to cells	*fedA*	F: GCTAATCAAGGGGGAGTGGC R: ACAGTGCTATTCGACGCCTT	110	This study
LT toxin production	*eltA*	F: TTGGTGATCCGGTGGGAAAC R: AGGAGGTTTCTGCGTTAGGTG	185	[20]
	*eltB*	F: CACGGAGCTCCCCAGACTAT R: GCCTGCCATCGATTCCGTAT	105	
STa toxin production	*estA*	F: CAACTGAATCACTTGACTCTT R: TTAATAACATCCAGCACAGG	158	
STb toxin production	*estB*	F: TGCCTATGCATCTACACAA R: CTCCAGCAGTACCATCTC	113	
Quorum sensing	*luxS*	F: CAGTGCCAGTTCTTCGTTGC R: TGAACGTCTACCAGTGTGGC	116	
Resistance to colistin	*MCR1*	F: GGGCCTGCGTATTTTAAGCG R: CATAGGCATTGCTGTGCGTC	184	This study
	*phoP*	F: CTGGTATTAACCGCCCGTGA R: GCGAAATGACCTGTGAAGCC	160	
	*pmrB*	F: ATCTGGAACTGCTGGCGAAA R: GAAAATGACTGTCCGGCACG	121	
	*pmrC*	F: GTACCGTGCATGTTCTCGGA R: CGCCATCGTTGTCATTCCAC	127	
	*pmrE*	F: TCATCATCGCCACTCCAACC R: GCGGTAAAACCAACGGGAAC	145	
Resistance to tetracyclines	*tetA*	F: GCTGTTTCCTTTTGCCGGAG R: TGAAGAAGACCGCCATCAGG	132	This study
	*tolC*	F: ACGAAGTGACCGCACGTAAT R: AGCGACAGGTTGCGTTTTTC	169	
	*emrK*	F: GCACAAAATGCGACAGGGAA R: GCATCTCGGCAATGTCTTCG	172	
	*emrY*	F: TGGGAGTCGACCTCAGAGAA R: GTCCGGCCCATATCGCATTA	154	

^1^ Ref = reference.

## Data Availability

The data presented in this study are available on request from the corresponding author.

## Supplementary Materials

The following supporting information can be downloaded at https://www.mdpi.com/article/10.3390/antibiotics11081073/s1. Figure S1: Effects of tetracycline (64 mg/L, TET) or a combination of tetracycline and thymol (MIC/4, 0.47 mM, TET+THY) on the expression of the adhesion-related *fedA* virulence gene in ETEC 95 and ETEC 97. The results were split and presented per individual strain. Data are expressed as the means of the three technical replicates of the two studied strains, with the SEM reported as vertical bars. For each gene, data were analyzed with one-way ANOVA with Tukey’s multiple comparison test; superscript letters (a, b) indicate significant differences among groups (*p* < 0.05).
