# Supplementary material for: Thymol as an Adjuvant to Restore Antibiotic Efficacy and Reduce Antimicrobial Resistance and Virulence Gene Expression in Enterotoxigenic Escherichia coli Strains
Source: Antibiotics (Basel). 2022 Aug 8;11(8):1073. doi: 10.3390/antibiotics11081073 (PMC9404878; doi:10.3390/antibiotics11081073)
Supplement: Supplementary file 1 [file antibiotics-11-01073-s001.zip › antibiotics-1866115-supplementary (2).pdf]

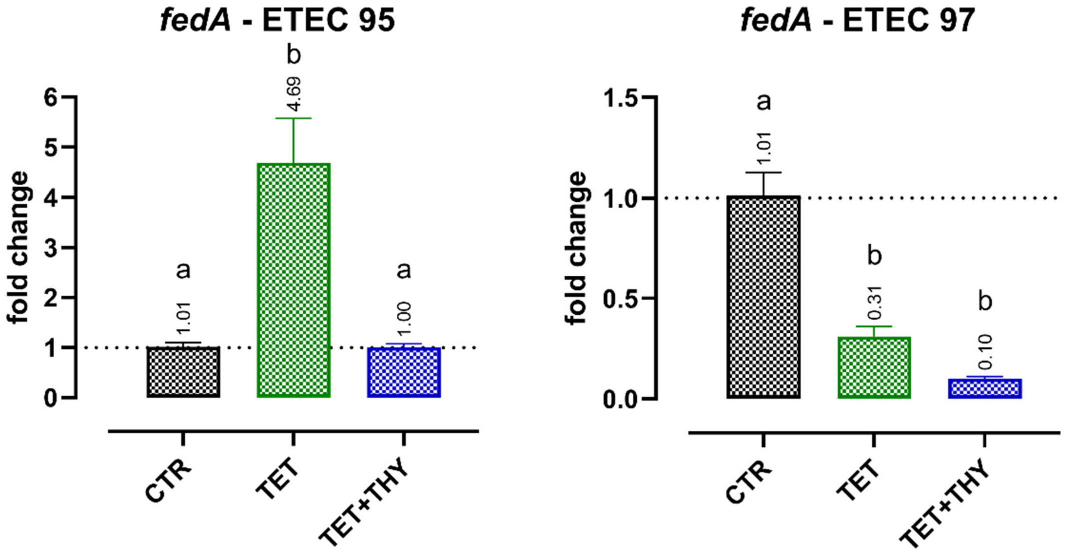

Figure S1. Effects of tetracycline (64 mg/L, TET) or a combination of tetracycline with thymol (MIC/4, 0.47 mM, TET+THY) on the expression of the adhesion-related *fedA* virulence gene in ETEC 95 and ETEC 97. The results were split and presented per individual strain. Data are expressed as the means of the three technical replicates of the two studied strains, with the SEM reported as vertical bars. For each gene, data were analyzed with one-way ANOVA with Tukey multiple comparison test; superscript letters (a, b) indicate significant differences among the groups ($p < 0.05$).
